# Construction of a machine learning-based risk prediction model for acute stroke in maintenance hemodialysis patients

**DOI:** 10.7717/peerj.21894

**Published:** 2026-11-11

**Authors:** Jinping Ying, Genlian Cai, Linglin Chen, Yujiao Zhang, Rui Zhang, Fei Han, Ping Zhang

**Affiliations:** 1 Department of Nursing, The First Affiliated Hospital, Zhejiang University School of Medicine, Hangzhou, Zhejiang, China; 2 Kidney Disease Center, The First Affiliated Hospital, Zhejiang University School of Medicine, Hangzhou, Zhejiang, China

**Keywords:** Acute stroke, Hemodialysis, Machine learning, Risk prediction

## Abstract

**Background:**

Acute stroke represents a significant complication in patients undergoing maintenance hemodialysis (MHD) and is associated with increased mortality rates in this population. This study aimed to construct a prediction model for acute stroke in these patients by using seven machine learning algorithms.

**Methods:**

This study included 1,100 patients receiving MHD at the First Affiliated Hospital, Zhejiang University School of Medicine, between January 1, 2015, and December 31, 2024. The patients were divided into training (*n* = 770) and test (*n* = 330) sets at a 7:3 ratio. Following feature selection using least absolute shrinkage and selection operator (LASSO) regression, predictive models were developed using seven machine learning algorithms. Model performance was evaluated using the area under the receiver operating characteristic (ROC) curve (AUC), sensitivity, specificity, and the Brier score. SHapley Additive exPlanations (SHAP) were used to quantify the contribution of individual features to model predictions. A web-based prediction tool was subsequently developed using the Shiny framework in R.

**Results:**

The results showed that the extreme gradient boosting (XGBoost) model performed the best in the test set, with an AUC of 0.847 (95% confidence interval (CI) [0.791–0.902]), sensitivity = 0.73, specificity = 0.83, F1-score = 0.61 and a Brier score of 0.115. The SHAP analysis identified six most important predictors, which were ranked in the descending order as follows: pre-dialysis systolic blood pressure (SBP), serum albumin level, uric acid level, hemoglobin level, C-reactive protein level, and a history of coronary heart disease.

**Conclusions:**

We successfully developed an interpretable XGBoost-based model that effectively predicts acute stroke risk in MHD patients.

## Introduction

Hemodialysis is the primary renal replacement therapy for patients with end-stage renal disease (Himmelfarb et al., 2020). Although hemodialysis effectively removes metabolic waste and harmful substances from the body, it cannot fully replicate the physiological functions of the kidneys. As the duration of dialysis increases, various complications emerge, significantly impacting the long-term survival of maintenance hemodialysis (MHD) patients (Massy & Drueke, 2022). Among these complications, stroke is particularly severe, with an incidence rate of 1.0% to 3.3% per year—5 to 10 times higher than that in the general population (Findlay et al., 2015). Even after treatment, more than 70% of stroke patients still have neurological disabilities after discharge, and the mortality rate within 1 year is still as high as 8.64% (Rost et al., 2022). Moreover, compared with MHD patients without stroke, those who have experienced stroke have a markedly greater prevalence of cognitive dysfunction, accompanied by a substantial decline in quality of life, extended duration of hospital stay, and significantly increased medical expenditures (Lan et al., 2019). Given its high incidence, recurrence, disability, and mortality rates, stroke is the second leading cause of death in MHD patients (United States Renal Data system, 2020). Therefore, how to identify individuals at high risk of stroke among patients undergoing MHD accurately and early and promptly implement targeted intervention measures has become an important issue that clinical medical workers urgently need to address.

Machine learning (ML) has become an important data analysis tool in medical research. Compared with traditional statistical methods, ML can be used to capture nonlinear relationships and complex interactions among multiple predictive variables, accurately identify disease risk factors, and enable early prediction and diagnosis (Greener et al., 2022). ML has become an important data analysis tool in medical research and is widely used in disease risk prediction, including the exploration of stroke prediction models in the general population and the prediction of acute ischemic stroke in patients after hip surgery. Six and three machine learning algorithms were developed in two studies (Zhi et al., 2024; Fu et al., 2025), and the constructed models demonstrated good performance and strong generalizability capabilities.

Nevertheless, the application of machine learning in predicting stroke risk among MHD patients is still in its infancy, with relatively few studies conducted to date. Therefore, the aim of our study was to construct a precise, effective, and clinically applicable risk prediction model for acute stroke in MHD patients using machine learning algorithms. This model could facilitate early and rapid screening of patients with a high risk for acute stroke by clinicians, enabling personalized management measures to improve patient outcomes.

## Materials and Methods

### Study population

This study was designed and conducted in accordance with the transparent reporting of a multivariable prediction model for individual prognosis or diagnosis plus artificial intelligence (TRIPOD + AI) statement (File S1). This study included patients who underwent MHD and received treatment at the First Affiliated Hospital of Zhejiang University School of Medicine from 1 January 2015 to 31 December 2024.

We enrolled patients who were aged ≥18 years and had received hemodialysis treatment for ≥3 months. The exclusion criteria were patients with acute stroke prior to starting hemodialysis or missing key clinical information and patients who developed coronary heart disease after enrollment in the study.

A total of 1,100 MHD patients were eventually included and randomly divided into a training set and a test set at a ratio of 7:3. The training set was used for model construction, and the test set was used for model evaluation. The specific study flowchart is shown in Fig. 1. This study was approved by the Ethics Review Committee of the First Affiliated Hospital of the Zhejiang University School of Medicine ([2025B] IIT Ethics Approval No. 0242). Given that this was a retrospective study, the need to obtain informed consent was waived. The study was conducted in accordance with the Declaration of Helsinki.

**Figure 1:**
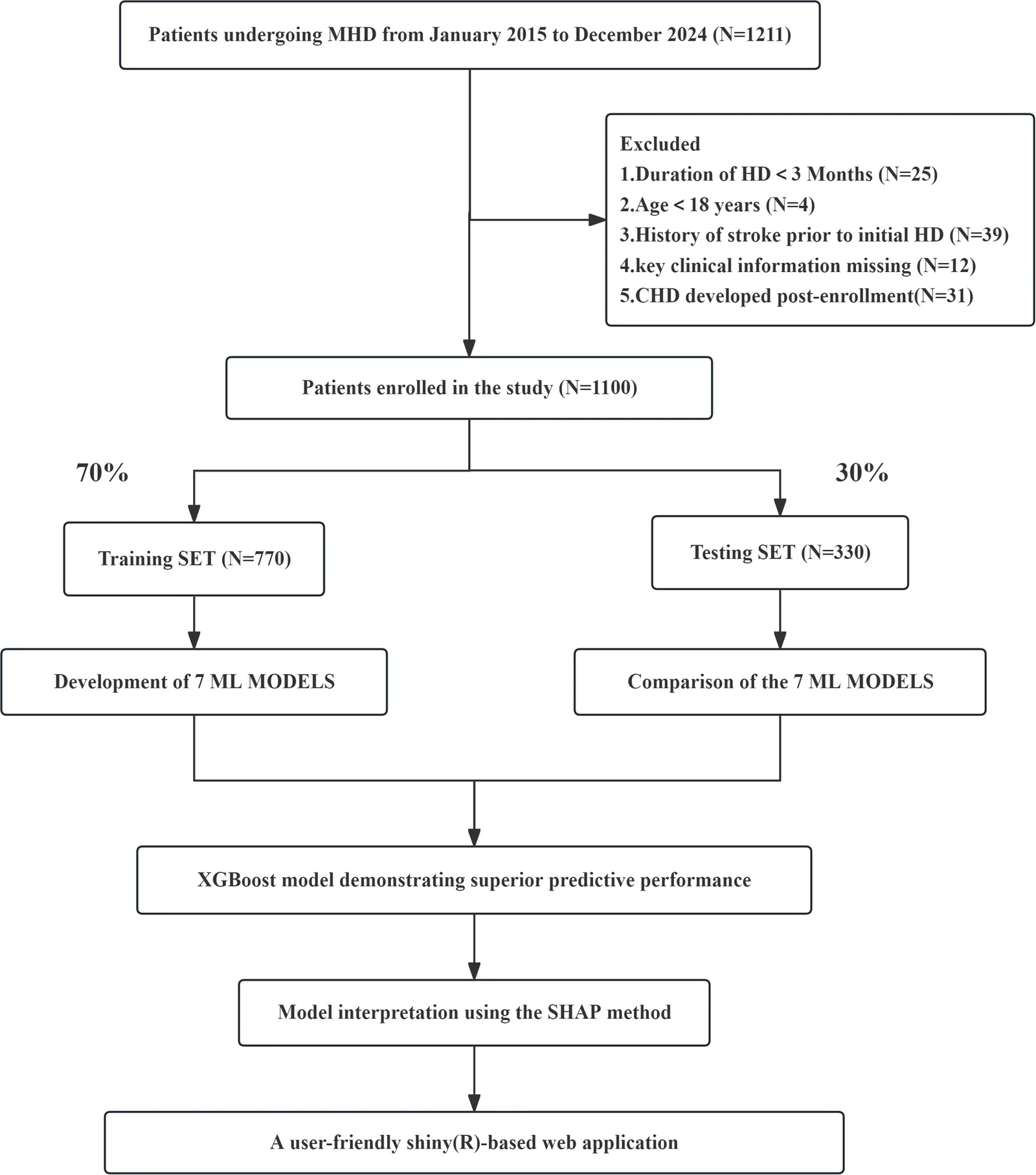
Study flowchart.

### Study variables and data preprocessing

In this study, on the basis of literature reviews (Hannedouche et al., 2016; Hsu et al., 2017; Matsushita et al., 2022; Park et al., 2022; Tsuneyoshi et al., 2021; Yeun et al., 2000) and expert opinions, we identified the following four categories of candidate predictors. Sociodemographic data included sex, age, history of smoking, history of alcohol consumption, and body mass index (BMI). BMI was calculated as dry weight (kg) divided by height squared (m²). The comorbidities included history of diabetes, history of coronary heart disease (CHD), and history of atrial fibrillation. A history of CHD refers to situations in which, prior to the first data collection after enrollment, percutaneous coronary intervention (PCI), coronary artery bypass grafting (CABG), or coronary artery stenosis ≥50% diagnosed by coronary angiography. Dialysis-related data included duration of dialysis, interdialytic weight gain rate, pre-dialysis systolic blood pressure (SBP), and pre-dialysis diastolic blood pressure (DBP). After ensuring the patient is in a supine position and has been at rest for 5 min, with both the back and arms relaxed, blood pressure should be measured in the arm without a fistula using the sphygmomanometer integrated into the dialysis machine. The laboratory indicators included hemoglobin, C-reactive protein (CRP), serum albumin, uric acid, white blood cells, the platelet–lymphocyte ratio (PLR), triglycerides, non-high-density lipoprotein cholesterol (non-HDL-C), serum sodium, serum phosphorus, serum calcium, fibrinogen, parathyroid hormone (PTH) and the urea clearance index (Kt/V). In this study, Kt/V was determined utilising the internationally recognised Daugirdas II formula, which is specified as follows: Kt/V = −ln(R−0.008t) + (4−3.5R) × UF/W. All clinical and laboratory data were extracted from the most recent routine assessments performed at the Index Date. Blood samples were randomly collected before dialysis at the beginning of the week.

As all study participants were recruited from the same region, where dietary patterns are relatively uniform, the influence of dietary factors on laboratory indicators is minimal.

To ensure the accuracy and reliability of the data, research materials were collected by two investigators from the hemodialysis electronic medical records system and verified by another investigator. Prior to statistical analysis, data cleaning was performed. Missing or abnormal data were checked to ensure accuracy. Values considered biologically implausible or inconsistent with predefined clinical plausibility criteria were recoded as missing values and subsequently handled using multiple imputation. Data collectors did not participate in subsequent data analysis.

The endpoint was defined as the date of the first acute stroke event or research deadline, which was either 31 December 2024.

Stroke was defined as the first occurrence of neurological deficits lasting >24 h due to cerebrovascular causes (Wu et al., 2023), including both ischemic stroke (ICD-10: I63) and hemorrhagic stroke (ICD-10: I60–I62), while excluding transient ischemic attack (TIA). All diagnoses were confirmed by neurologists based on computed tomography (CT) or magnetic resonance imaging (MRI) findings from the hospital’s electronic medical records.

In this study, variables within the dataset with more than 20% missing values, such as household income and B-type natriuretic peptide, were excluded from the analysis. In contrast, variables including BMI, uric acid, Kt/V, PTH, and fibrinogen exhibited less than 5% missing values. For these variables, a 10-fold multiple imputation method was utilized to address the missing data. The imputed dataset that simultaneously minimized both the Akaike Information Criterion (AIC) and Bayesian Information Criterion (BIC) was selected for subsequent statistical analyses (Noghrehchi et al., 2021). For the training set, we used least absolute shrinkage and selection operator (LASSO) regression for feature selection. We performed 10-fold cross-validation and chose the lambda.1se value (the smallest λ value within one standard error of the minimum) as the optimal λ. Features with nonzero coefficients were then selected for model construction.

### Model performance evaluation

On the basis of the features selected by LASSO regression, we further used seven machine learning algorithms to build models, including logistic regression (LR), decision tree (DT), random forest (RF), support vector machine (SVM), extreme gradient boosting (XGBoost), light gradient boosting machine (LGBM), and k-nearest neighbor (KNN). Model performance was evaluated using indicators such as the area under the receiver operating characteristic (ROC) curve (AUC), sensitivity, specificity, accuracy, F1-score, and Brier score to compare the predictive performance of the models in the test set. The Brier score is a commonly used metric for evaluating the calibration of probability predictions (Huang et al., 2020). The DeLong test was used to determine whether the AUC values of the different models were significantly different. This test is a nonparametric method specifically designed for comparing correlated ROC curves without assuming underlying data distributions (Wynants et al., 2019).

### Model interpretation and web application

The SHAP method was used to interpret the results of the optimal prediction model and rank the importance of the input features. A web application based on the Shiny R framework was developed to deploy the best-performing prediction model. To mitigate input errors and subsequent prediction bias, categorical variables, including the history of coronary heart disease, were constrained to binary options, with “0” indicating no history and “1” indicating a history. For continuous variables, alerts were activated upon the entry of abnormal values. To improve interpretability, the application presents risk levels segmented by terciles of predicted probabilities.

### Statistical analysis

Data analysis was performed using R (version 4.4.2; R Core Team, 2024) and DCPM (V4.01, Jingding Medical Technology Co., Ltd., Xiamen, Fujian, China) programming software, including packages such as pmsampsize, CBCgrps, glmnet, rpart, RF, XGBoost, e1071, kknn, and lightgbm. Normally distributed measurement data are expressed as the means ± standard deviations, and intergroup comparisons were made using *t* tests. Nonnormally distributed measurement data are expressed as medians and quartiles, and intergroup comparisons were made using the Mann‒Whitney U test. Count data are expressed as frequencies and percentages (%), and intergroup comparisons were made using chi-square tests or Fisher’s exact probability tests. A *P* value < 0.05 was considered for statistical significance.

## Results

### Patient baseline characteristics

A total of 1,100 MHD patients were included in this study. The research dataset was randomly divided into a training set and a test set at a ratio of 7:3. In the training set, the median age of the patients was 62 years (IQR 51–71 years), with 471 male patients and 299 female patients. A total of 116 patients had acute stroke, with an incidence rate of 15.1%. In the test set, the median age was 61.5 years (IQR 50–70 years), with 205 male patients and 125 female patients. A total of 67 patients had acute stroke, with an incidence rate of 20.0%. No significant differences were found in the baseline characteristics between the training and test sets (Table 1).

**Table 1:** Baseline characteristics of the patients in the training and test sets.

Variables	Total (*n* = 1,100)	Test set (*n* = 330)	Training set (*n* = 770)	*P* value	Statistic
Sex, *n* (%)				0.818^b^	0.053
Female	424 (39)	125 (38)	299 (39)		
Male	676 (61)	205 (62)	471 (61)		
Age (year, median (Q1, Q3))	62 (51, 71)	61.5 (50, 70)	62 (51, 71)	0.317^c^	122,218
History of smoking, *n* (%)	175 (16)	55 (17)	120 (16)	0.719^b^	0.129
History of drinking, *n* (%)	78 (7)	23 (7)	55 (7)	1^b^	0
BMI (kg/m2, median (Q1, Q3))	21.8 (19.5, 24.3)	21.85 (19.4, 24.37)	21.75 (19.6, 24.3)	0.904^c^	127,632.5
History of diabetes, *n* (%)	328 (30)	102 (31)	226 (29)	0.656^b^	0.199
History of CHD, *n* (%)	104 (9)	24 (7)	80 (10)	0.132^b^	2.27
History of atrial fibrillation, *n* (%)	60 (5)	22 (7)	38 (5)	0.311^b^	1.028
Dialysis duration (month, median (Q1, Q3))	45 (24, 102)	45 (28, 84.75)	45 (23, 106.75)	0.942^c^	127,404
IDWG (%, median (Q1, Q3))	3.23 (2.29, 4.41)	3.34 (2.25, 4.54)	3.21 (2.33, 4.34)	0.573^c^	129,770
Pre-dialysis SBP (mmHg, median (Q1, Q3))	136 (121, 151)	136 (123, 151)	136 (121, 150.75)	0.671^c^	129,102
Pre-dialysis DBP (mmHg, Mean ± SD)	76.74 ± 14.45	77.28 ± 14.39	76.51 ± 14.47	0.419^a^	0.809
Hemoglobin (mg/L, median (Q1, Q3))	116 (107, 123)	115 (105.25, 123)	117 (107, 124)	0.069^c^	118,277
CRP (mg/L, median (Q1, Q3))	2.5 (0.99, 6)	2.61 (0.88, 5.99)	2.46 (1, 6.01)	0.977^c^	126,908.5
Serum albumin (g/L, median (Q1, Q3))	40 (37.9, 42)	40.2 (38, 42.08)	39.9 (37.8, 42)	0.961^c^	126,815.5
Uric acid (μmol/L, median (Q1, Q3))	421.5 (358, 488)	410 (349.25, 475)	424 (362.25, 490)	0.065^c^	118,124
WBC (×10^9^/L, median (Q1, Q3))	5.92 (4.85, 7.44)	5.97 (4.81, 7.47)	5.9 (4.88, 7.44)	0.844^c^	126,098.5
PLR, median (Q1, Q3)	176.9 (131.85, 230.61)	177.17 (129.61, 241.24)	176.84 (132.64, 228.03)	0.579^c^	129,732
Triglycerides (mmol/L, median (Q1, Q3))	1.73 (1.17, 2.63)	1.62 (1.05, 2.38)	1.78 (1.19, 2.75)	0.008^c^	114,335
Non-HDL-C (mmol/L, median (Q1, Q3))	2.52 (1.96, 3.23)	2.47 (1.88, 3.21)	2.54 (2.01, 3.25)	0.314^c^	122,191.5
Serum sodium (mmol/L, median (Q1, Q3))	139 (136, 141)	138 (136, 141)	139 (137, 141)	0.579^c^	124,380.5
Serum Phosphorous (mmol/L, median (Q1, Q3))	1.62 (1.33, 1.97)	1.59 (1.28, 1.97)	1.63 (1.35, 1.97)	0.205^c^	120,936.5
Serum Calcium (mmol/L, median (Q1, Q3))	2.23 (2.12, 2.35)	2.22 (2.12, 2.35)	2.23 (2.11, 2.36)	0.815^c^	125,917.5
Fibrinogen (g/L, median (Q1, Q3))	3.39 (2.94, 3.94)	3.42 (2.94, 4.02)	3.38 (2.94, 3.93)	0.522^c^	130,141.5
PTH (pg/mL, median (Q1, Q3))	203 (120.5, 307.55)	214 (128.77, 325.6)	199.5 (114.25, 302)	0.124^c^	134,475.5
Kt/V, median (Q1, Q3)	1.51 (1.33, 1.7)	1.49 (1.34, 1.7)	1.52 (1.33, 1.7)	0.389^c^	122,894.5

**Notes:**

BMI, body mass index; CHD, coronary heart disease; IDWG, rate of interphase dialysis weight gain; CRP, C-reactive protein; WBC, white blood cells; Non-HDL-C, non-high-density lipoprotein cholesterol; PTH, parathyroid hormone; PLR, platelet/lymphocyte ratio; Kt/V, urea clearance index; IQR, interquartile range; SD, standard deviation.

a Student’s t test.

b Chi-square test.

c Mann–Whitney U test.

### Feature selection

To identify the most relevant predictors for acute stroke in MHD patients, LASSO regression was applied to the preprocessed dataset. Tenfold cross-validation was employed to determine the optimal regularization parameter (λ) for the model. Two λ values were calculated: lambda .min (0.01347347) and lambda.1se (0.03749072). Given that lambda.1se provides a more robust model by balancing the trade-off between model complexity and prediction accuracy, it was selected as the optimal parameter. Ultimately, six features with nonzero coefficients were identified, including pre-dialysis SBP, serum albumin, uric acid, hemoglobin, CRP and history of CHD (Figs. 2A and 2B). These features were subsequently used as predictive variables to construct the prediction model.

**Figure 2:**
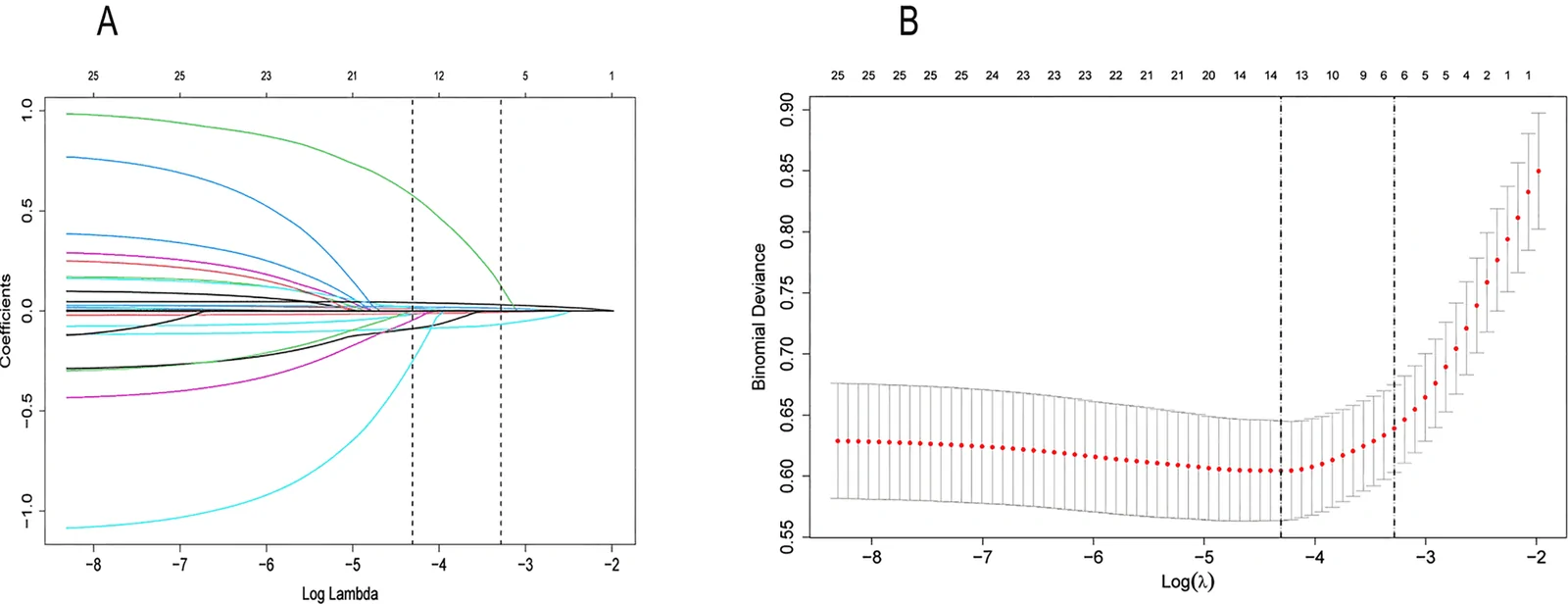
(A) LASSO path plot. (B) LASSO tenfold cross-validation error plot.

### Comparison of prediction model performance

In the training set, the AUC values of each model ranged from 0.865 to 1.000 (Fig. 3A). In the test set, the AUC values ranged from 0.726 to 0.847 (Fig. 3B). In the test set, the AUC values of each model in descending order are as follows: XGBoost (AUC = 0.847), LGBM (AUC = 0.832), LR (AUC = 0.831), SVM (AUC = 0.831), RF (AUC = 0.827), KNN (AUC = 0.738), and DT (AUC = 0.726).

**Figure 3:**
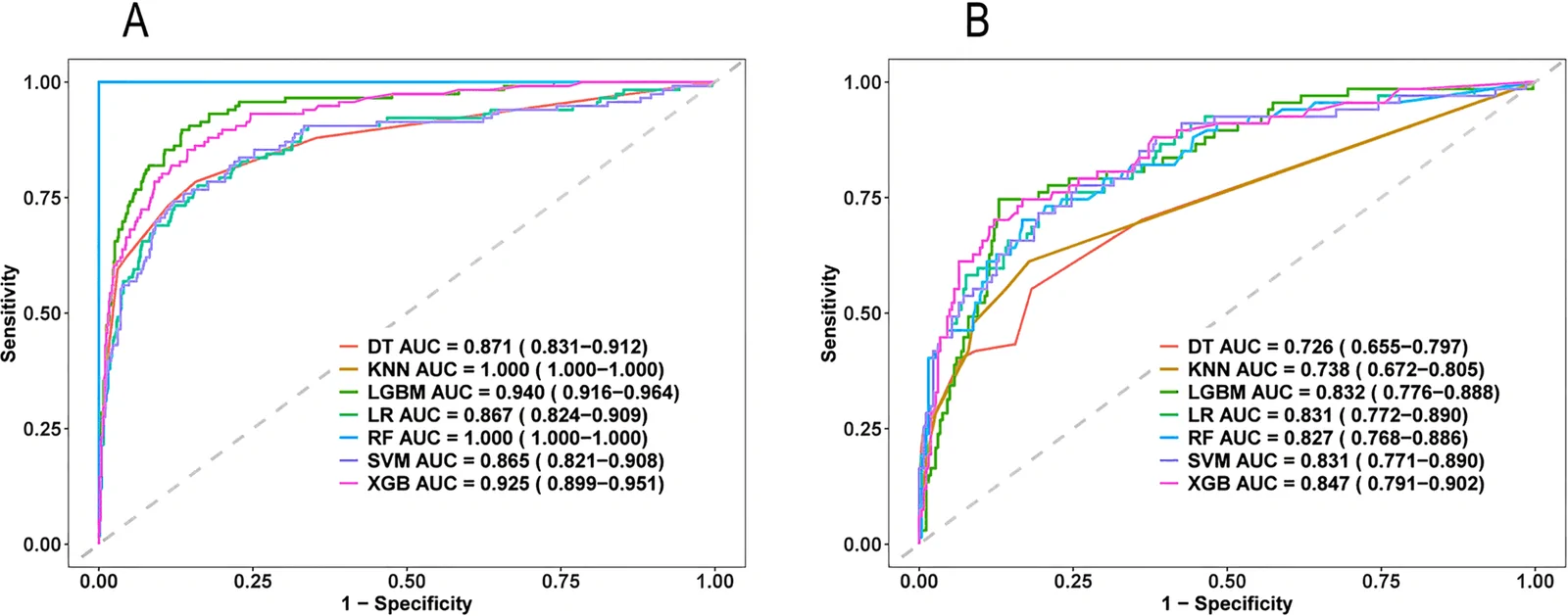
(A) Training set AUC (B) Test set AUC.

According to the DeLong test, the AUC of the XGBoost model in the test set was not significantly different from those of the LR, DT, RF, SVM, and LGBM models (*P* > 0.05), but it was significantly greater than that of the KNN model (*P* < 0.05). In the test set, the XGBoost model achieved a sensitivity of 0.73, specificity of 0.83, F1-score of 0.61, and Brier score of 0.115 (Table 2). These metrics indicate that the XGBoost model outperformed the other models in terms of predictive performance, demonstrating good efficacy and robustness. Meanwhile, Decision Curve Analysis (DCA) pointed out that, compared with other models, the XGBoost model showed better clinical benefits than both the “treat All” and “treat none” strategies (Fig. 4B).

**Table 2:** Performance of the prediction models for acute stroke in the training and test sets.

Model	AUC	Sensitivity	Specificity	Accuracy	PPV	NPV	F1-score	Brier
Training set								
LR	0.867	0.78	0.84	0.87	0.46	0.95	0.58	0.0808
DT	0.871	0.78	0.93	0.91	0.59	0.97	0.67	0.0718
RF	1	1	1	1	1	1	1	0.0118
XGBoost	0.925	0.85	0.86	0.86	0.51	0.97	0.64	0.0717
SVM	0.865	0.76	0.86	0.85	0.49	0.95	0.6	0.0817
KNN	1	1	1	1	1	1	1	0.0174
LGBM	0.94	0.9	0.86	0.87	0.54	0.98	0.68	0.0620
Test set								
LR	0.831	0.66	0.83	0.83	0.49	0.9	0.56	0.1119
DT	0.726	0.6	0.86	0.82	0.4	0.93	0.48	0.1397
RF	0.827	0.45	0.95	0.85	0.71	0.87	0.55	0.1145
XGBoost	0.847	0.73	0.83	0.81	0.53	0.92	0.61	0.1152
SVM	0.831	0.66	0.84	0.80	0.51	0.91	0.57	0.1116
KNN	0.738	0.42	0.92	0.82	0.57	0.86	0.48	0.1438
LGBM	0.832	0.75	0.86	0.84	0.58	0.93	0.65	0.1272

**Note:**

LR, logistic regression; SVM, support vector machine; DT, decision tree; RF, random forest; XGBoost, extreme gradient boosting; LGBM, light gradient boosting machine; KNN, k-nearest neighbors; PPV, positive predictive value; NPV, negative predictive value; AUC, area under the receiver operating characteristic curve.

**Figure 4:**
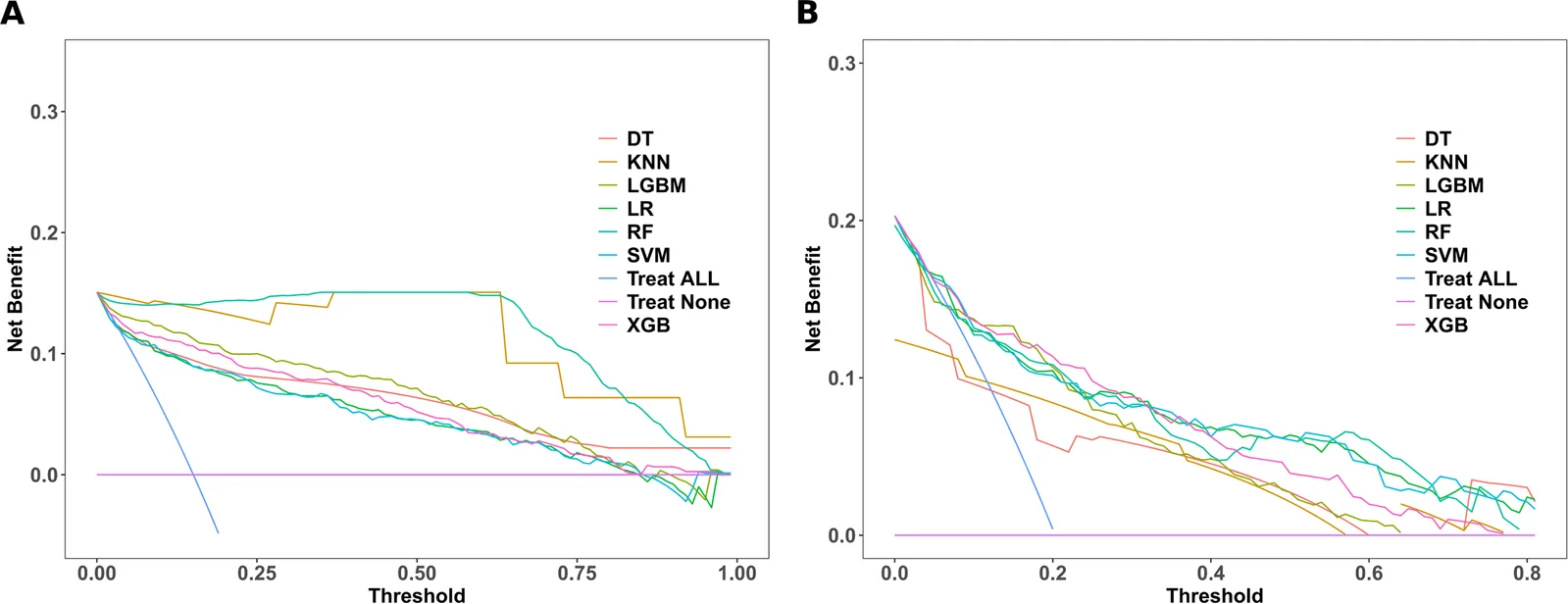
(A) Training set DCA (B) Test set DCA.

### Model interpretation and web application

The XGBoost model uses the SHAP mean method for feature interpretation. The features were ranked by importance in descending order as follows: pre-dialysis SBP, serum albumin, uric acid, hemoglobin, CRP, and history of CHD. pre-dialysis SBP had the most significant effect on the model output, as indicated by the longest bar in the feature importance plot (Fig. 5A). In contrast, a history of CHD had a relatively small impact, with a shorter bar length.

**Figure 5:**
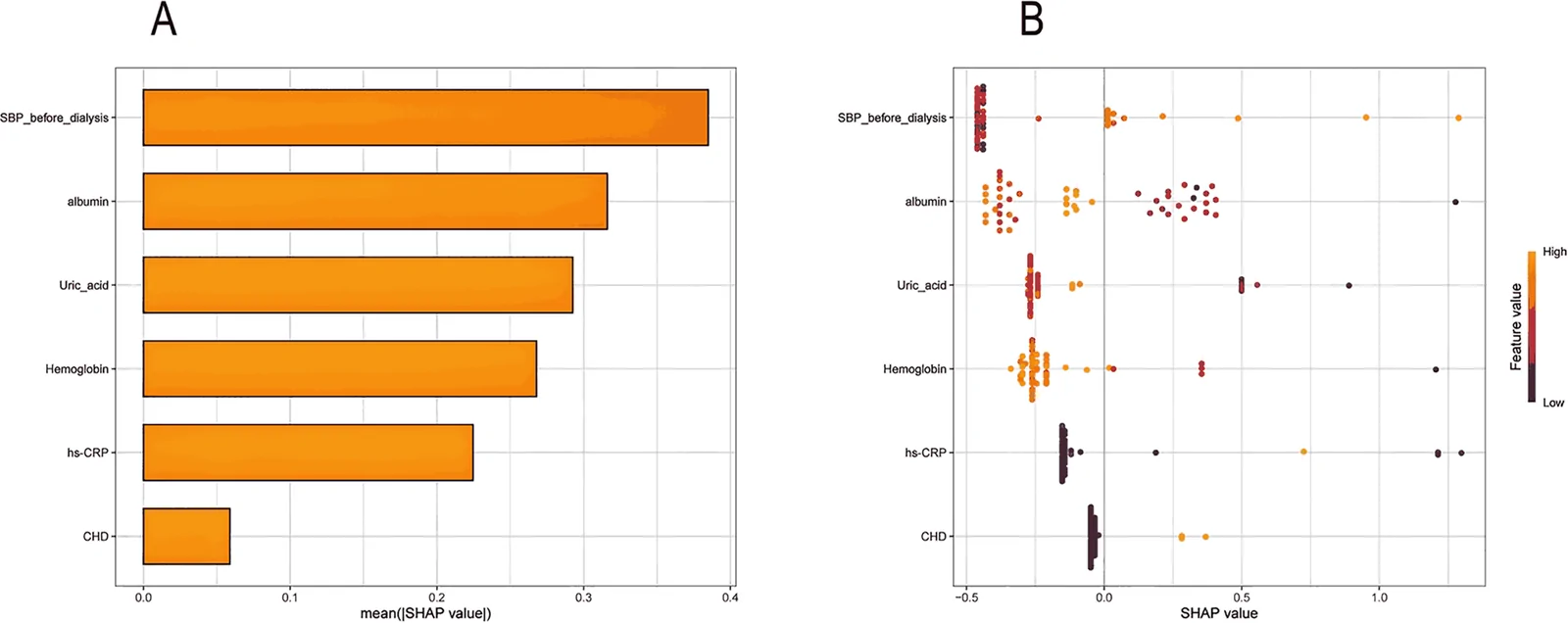
(A) SHAP summary bar chart (B) SHAP summary dot plot. SBP, systolic blood pressure; CRP, C-reactive protein; CHD, coronary heart disease.

To further analyze the direction and magnitude of each feature’s influence on the model’s predictions, a SHAP summary dot plot was generated (Fig. 5B). In this plot, positive and negative SHAP values represent the positive and negative impacts of feature values on the model’s predictions, respectively. Yellow dots indicate high feature values, whereas purple dots indicate low feature values. The visualization results indicate that there were significant differences in the impacts of various features on model predictions. Moreover, the levels of feature values show specific correlation patterns with model outputs, revealing certain regularities overall. For example, when the pre-dialysis SBP is relatively high, its SHAP values are mainly distributed in the positive region, suggesting that high pre-dialysis SBP contributes to the risk of stroke. For indicators such as serum albumin and uric acid, the different contributions of their high and low values to model predictions can be observed on the basis of the color gradients in the dot plots.

Local interpretability is assessed by calculating and displaying the specific contributions of each feature to the prediction of individual samples. In Fig. 6, red labels indicate negative contributions, whereas yellow labels indicate positive contributions. The numbers on the labels precisely reflect the specific contribution values of each feature. For this patient, the pre-dialysis SBP was 97 mmHg, the serum albumin level was 38.6 g/L, the uric acid concentration was 537 μmol/L, the hemoglobin concentration was 102 mg/L, the CRP concentration was 0.9 mg/L, and there was no history of CHD. The SHAP value decomposition of each feature for the acute stroke prediction model is shown. The cumulative result of these contribution values is the final prediction value, which is −1.91. This indicates that the model considers the patient’s stroke risk to be below the decision threshold, suggesting a low likelihood of stroke occurrence for this patient.

**Figure 6:**
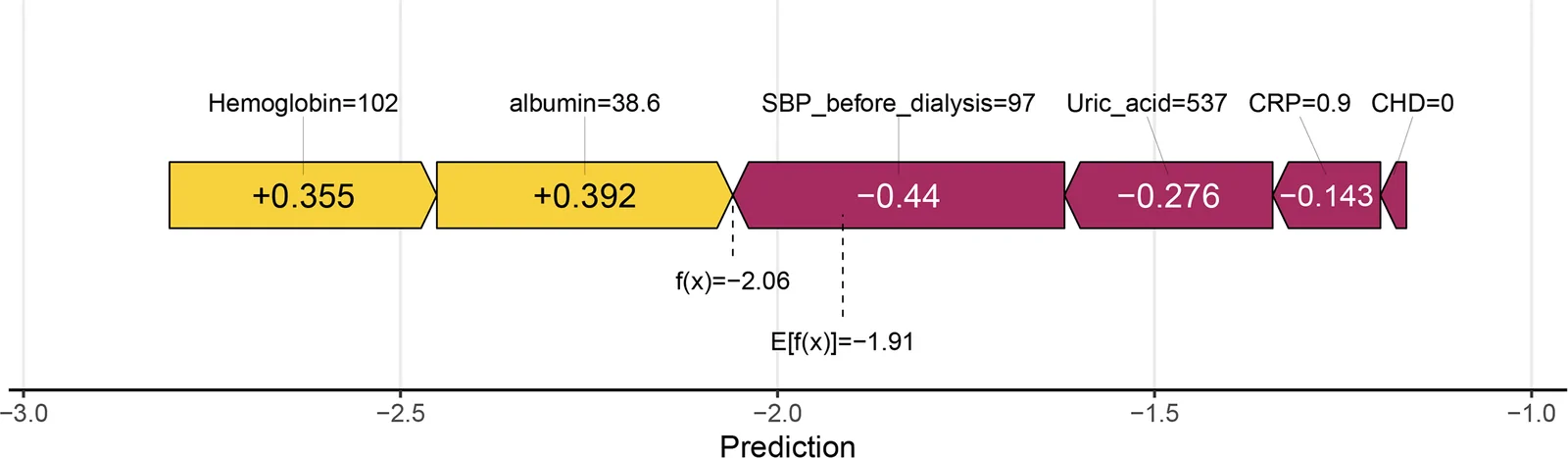
SHAP waterfall plot. SBP, systolic blood pressure; CRP, C-reactive protein; CHD, coronary heart disease.

The web application developed on the basis of the XGBoost model in this study allows users to input the actual values of the six features required by the model (pre-dialysis SBP, serum albumin, uric acid, hemoglobin, CRP, and history of CHD). It then automatically calculates and outputs the probability of acute stroke occurrence in hemodialysis patients, facilitating their use by clinical medical staff. For example, for a patient with a pre-dialysis SBP of 196 mmHg, a hemoglobin concentration of 108 mg/L, a history of CHD, an CRP of 20 mg/L, a serum albumin concentration of 32.6 g/L, and a uric acid concentration of 486 μmol/L, the patient has a stroke risk probability of 0.655, categorizing them as a medium-risk patient (Fig. 7), indicating that targeted interventions should be provided for this patient in clinical practice. The web application can be accessed online *via* the following link: https://xiaobai1234567.shinyapps.io/stroke_XGBoost/.

**Figure 7:**
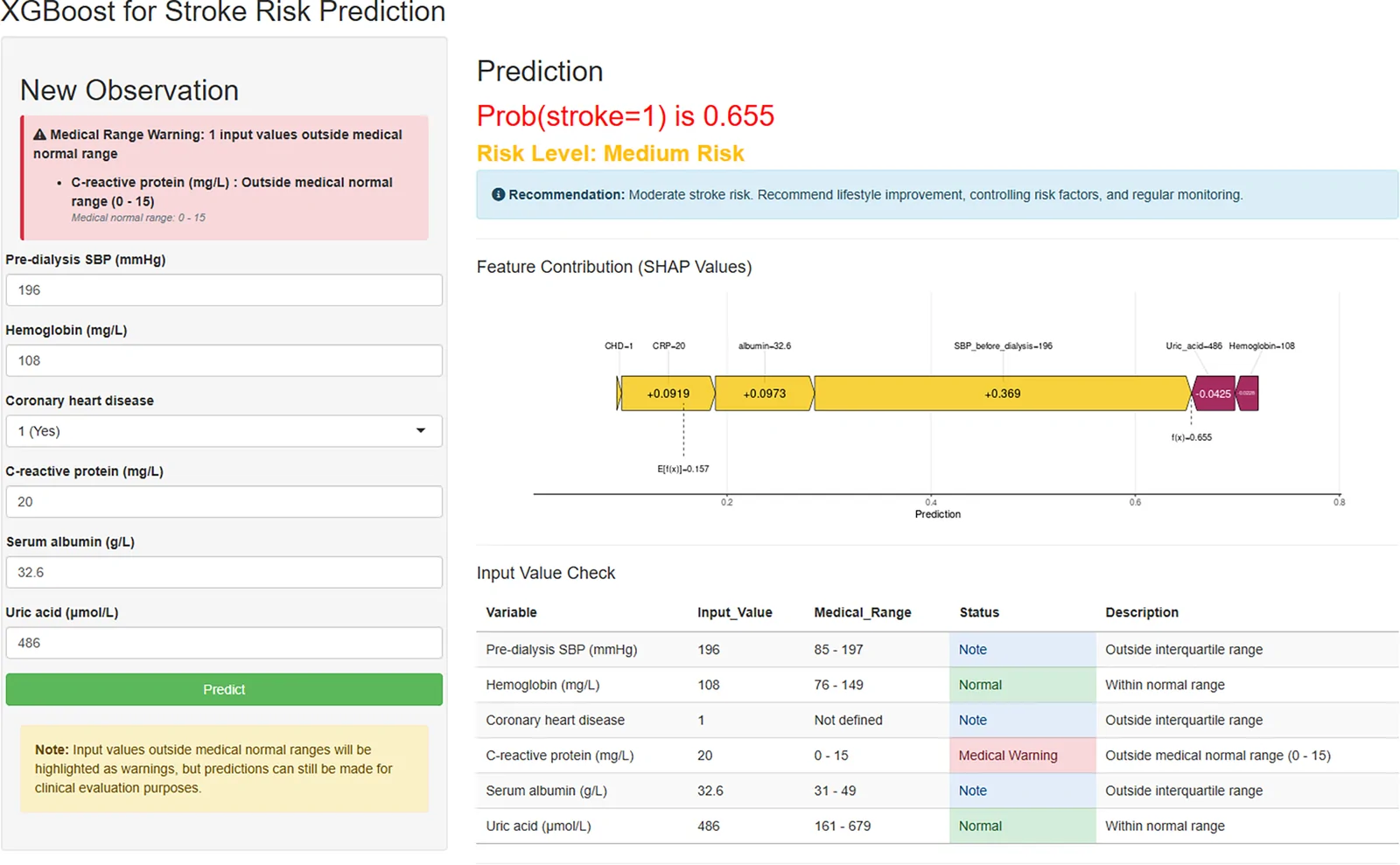
Clinical application interface. SBP, systolic blood pressure.

## Discussion

Innovatively, in this study, we used seven machine learning algorithms to construct a prediction model for acute stroke risk in maintenance hemodialysis patients. Through systematic comparisons, the XGBoost model demonstrated the best predictive performance, with a test set AUC of 0.847. Additionally, it exhibited favorable metrics such as sensitivity (0.73), specificity (0.83), F1-score (0.61), and Brier score (0.1152). Compared with previous studies, our model has significant advantages. For example, Zhou et al. (2024) reported internal and external validation AUCs of 0.768 and 0.732, respectively, for their ischemic stroke model based on logistic regression. Moreover, Yu-Huan, Guang-Yan & Yue-Fei (2023) achieved AUCs of only 0.732 and 0.624 for their intracerebral hemorrhage prediction model, which was based on blood pressure and PTH. The enhanced performance of our model may be attributed to the ability of machine learning algorithms to capture nonlinear relationships, taking into account the interactions and synergy effects among variables, as well as optimizing the process of variable selection.

To enhance the practicality and clinical applicability of the model, we developed a web-based calculator. This tool is easy to use; clinical medical staff can simply enter the actual values of key features on the web page to quickly assess the patient’s stroke probability in real time. This immediate feedback can support individualized intervention decisions, thereby helping clinical medical staff create personalized management plans, effectively reduce the incidence of stroke, and improve patient outcomes.

Moreover, the six key feature variables identified in this study are objective indicators measured by reliable methods, and their acquisition process is simple, which endows the constructed model with broad clinical applicability. For example, the SHAP feature importance ranking shows that pre-dialysis SBP ranks first, which is highly consistent with the clinical evidence that hypertension is a core risk factor for stroke. A study by Hannedouche et al. (2016) demonstrated that for every 10 mmHg increase in pre-dialysis SBP, the risk of stroke increases by 1.15 times. The mechanism by which hypertension promotes stroke occurrence is mainly attributed to the activation of angiotensin II and the subsequent increase in reactive oxygen species production and phospholipase C activation (Alkahtani, 2022). For MHD patients, chronic hypertension subjects vessel walls to excessive pressure, causing endothelial damage, promoting the formation and progression of atherosclerosis, and increasing the risk of cerebral vessel rupture and occlusion (Bucci et al., 2024). This finding not only strengthens the scientific basis of the model but also provides a clear direction for clinical practice.

Serum albumin ranked second. Hypoalbuminemia, a marker of malnutrition and systemic inflammation in MHD patients, may reduce physiological reserve and impair endothelial function through diminished antioxidant/anti-inflammatory properties, thereby promoting atherosclerosis and increasing stroke risk (Zhou et al., 2021; Bucci et al., 2024).

The relationship between uric acid and stroke risk in dialysis patients remains controversial. While elevated uric acid has been traditionally associated with increased cardiovascular risk through mechanisms such as endothelial dysfunction and oxidative stress (Zhu et al., 2025), some studies in MHD populations have suggested that higher uric acid may paradoxically reflect better nutrition or confer neuroprotection (Hsu et al., 2017). Our findings confirm its predictive value, though the bidirectional role requires further exploration.

Higher hemoglobin levels can increase blood viscosity, leading to hemodynamic disturbances and subsequently promoting thrombosis. Conversely, low hemoglobin may reflect more severe illness, poorer overall health status, or the presence of comorbidities (Lin et al., 2025), which can worsen prognosis, cause hypoxia in brain tissue, and thereby increase the risk of acute stroke (Esenwa & Elkind, 2016).

As a key inflammatory marker, CRP promotes plaque instability and hypercoagulability (Esenwa & Elkind, 2016; Akinyelure et al., 2022). In MHD patients, persistent microinflammation is common due to uremic milieu and dialysis-related factors, making CRP a particularly relevant predictor in this population (Yeun et al., 2000). Together with serum albumin and uric acid, CRP synergistically contributes to atherosclerosis and thrombogenesis (Zhou et al., 2021; Akinyelure et al., 2022; Huo et al., 2025; Zhu et al., 2025).

In this model, history of CHD ranked last in terms of feature importance. This phenomenon is primarily attributable to the frequent presentation of multiple complications in dialysis patients, which may exhibit complex interrelationships (Yu-Huan, Guang-Yan & Yue-Fei, 2023). Furthermore, the close relationship between CHD and hypertension affects the utility of CHD history for predicting the risk of stroke, as the predictive signal of CHD may be largely captured by other correlated features such as SBP and inflammatory markers.

Unlike previous studies (Aono et al., 2021; Tong et al., 2024), we did not find an association between a history of diabetes or serum calcium, serum phosphate, or PTH levels and the risk of stroke in hemodialysis patients. This may be related to the specific characteristics of the study population. For example, in this study, among the enrolled MHD patients, individuals with a history of diabetes typically received standardized management. This approach may have contributed to reducing the disparity in stroke risk between patients with a diabetic history and their non-diabetic counterparts within the cohort. Additionally, serum phosphate, calcium, and PTH levels were well controlled, which may have reduced the risk of stroke to some extent. Therefore, these findings do not exclude the possibility that these factors may have different impacts on other study populations.

Machine learning has shown great potential in the field of medical prediction and diagnosis, with many studies demonstrating its high predictive performance (Zhi et al., 2024; Li et al., 2023). XGBoost, with its dual advantages of regularization design at the algorithmic level and engineering optimization, has become the preferred choice for handling complex data in scenarios requiring high robustness (Chen, Guestrin & Association for Computing Machinery, 2016). Previous studies by Li et al. (2023) used XGBoost to predict the functional outcomes of spontaneous intracerebral hemorrhage, achieving an AUC of 0.979. Similarly, Guan et al. (2024) used XGBoost to predict new-onset atrial fibrillation in critically ill patients, with an AUC of 0.891 on the test set. These results indicate that XGBoost has good prediction performance in clinical settings. Our study also confirmed the advantages of the XGBoost algorithm.

Our study has several limitations. First, this was a single-center retrospective study, and the sample source was relatively limited, which may not fully represent a broader population. Second, the data did not account for social background factors, such as employment status, the income level, and the number of cohabiting family members, which may have introduced selection bias and thereby affected the accuracy of the results. Third, although we incorporated history of CHD as a predictor, we did not include concomitant medications (*e.g*., antiplatelets and statins) in the model, as patients with CHD are more likely to receive these agents, which may exert protective effects against stroke and thus potentially confound the observed association. Fourth, the enrolled patients had heterogeneous dialysis durations, reflecting different stages of maintenance hemodialysis. Although dialysis durations was considered as a candidate predictor during the model development process, it was not retained in the final models. Therefore, the potential impact of dialysis duration-related heterogeneity on stroke risk prediction cannot be completely excluded. Future research should adopt a prospective design, incorporate more relevant variables, and conduct multicentre, large-sample external validation to further verify the robustness and generalizability of the model, optimize its performance, and enhance its application value in clinical practice.

## Conclusions

In summary, this study developed a machine learning–based model to predict the risk of acute stroke in MHD patients. The model not only demonstrated robust predictive performance but also featured high interpretability, ease of use, and reliability, making it a valuable tool for predicting acute stroke risk in MHD patients in clinical practice.

## Supplemental Information

10.7717/peerj.21894/supp-1 Supplemental Information 1 Code.

10.7717/peerj.21894/supp-2 Supplemental Information 2 TRIPOD+AI checklist.

10.7717/peerj.21894/supp-3 Supplemental Information 3 Data Summary.

10.7717/peerj.21894/supp-4 Supplemental Information 4 Raw data.

10.7717/peerj.21894/supp-5 Supplemental Information 5 Categorical Data codebook.

## Additional Information and Declarations

### Data Availability

The following information was supplied regarding data availability:

The data and code are available in the Supplemental Files.
