# Supplementary material for: Construction of a machine learning-based risk prediction model for acute stroke in maintenance hemodialysis patients
Source: PeerJ. 2026 Nov 11;14:e21894. doi: 10.7717/peerj.21894 (PMC13769277; doi:10.7717/peerj.21894)
Supplement: Supplementary file 2 [file peerj-14-21894-s005.docx]

Assignment of categorical variables

Stroke：0 = No , 1=Yes

Gender：1=Female，2= Male

Smoking：0 = No , 1=Yes

Drinking：0 = No , 1=Yes

Diabetes	：0 = No , 1=Yes

CHD：0 = No , 1=Yes

Atrial_fibrillation：0 = No , 1=Yes
